# Ability of Adult *Dermacentor reticulatus* Ticks to Overwinter in the Temperate Climate Zone

**DOI:** 10.3390/biology9070145

**Published:** 2020-06-29

**Authors:** Zbigniew Zając, Katarzyna Bartosik, Joanna Kulisz, Aneta Woźniak

**Affiliations:** Chair and Department of Biology and Parasitology, Medical University of Lublin, Radziwiłłowska 11 St., 20-080 Lublin, Poland; katarzyna.bartosik@umlub.pl (K.B.); joanna.kulisz@umlub.pl (J.K.); aneta.wozniak@umlub.pl (A.W.)

**Keywords:** *Dermacentor reticulatus*, ticks, tick overwinter, tick activity, ticks and winter

## Abstract

*Dermacentor reticulatus* ticks, one of the most important vectors and reservoirs of tick-borne diseases in Europe, are widespread in the temperate climate zone and in some localities in the subtropical climate zone of the western Palaearctic region. These ticks occur in a large area characterised by a varied climate type, vegetation, and availability of potential hosts. Hence, they exhibit high ecological plasticity and adaptability to periodically adverse conditions. The aim of the present study was to investigate the ability of *D. reticulatus* adults to overwinter in the natural habitat. Specimens marked with a permanent oil marker on the festoons were placed in their natural habitats for the winter. Concurrently, tick survival in laboratory conditions at a temperature of 5 °C and 18 °C was assessed as a control. The groups were compared with each other by determination of the weight of fat bodies. In the field conditions, 67.9% females and 60.0% males survived the winter. There was no significant difference in the survival of ticks in the laboratory. A significantly lower fat body weight was found in the group of ticks overwintering in the field conditions and exhibiting questing activity between spring and late autumn during the following year. On the population scale, adult *D. reticulatus* ticks are able to survive the winter in temperate climate conditions at a level ensuring a further increase in their population size. In adverse weather conditions, ticks enter diapause, thus maximally reducing the utilisation of the content of their fat bodies. This facilitates long-term survival in the environment.

## 1. Introduction

The ornate cow tick *Dermacentor reticulatus* (Acari: Ixodida) is one of the most widespread tick species worldwide [1,2]. The distribution of *D. reticulatus* includes western Palaearctic regions with various types of temperate climate and some localities in the subtropical climate zone [2]. This tick species occurs in western Siberia and in the steppes of the European part of Russia and Ukraine, where the temperate continental climate is characterised by extremely high values (°C) of annual temperature (up to 45 °C) and low annual precipitation rates (mm) [3,4].

Ornate cow ticks are the most common species of the ixodid tick fauna in some regions of Central Europe located in the temperate climate zone, e.g., in eastern Poland [5] (average annual air temperature of ca. 10 °C) [4]. Large local *D. reticulatus* tick populations have also been reported in other countries of this region, e.g., the Czech Republic, Romania, Slovakia, and Hungary [6,7,8,9]. The dense occurrence range of *D. reticulatus* also covers France and the west of Germany [2], where the temperate oceanic climate is usually characterised by frostless winters and relatively high precipitation throughout the year [4]. Local islands of *D. reticulatus* populations have also been reported from the Iberian Peninsula, the Apennine Peninsula, and the Balkans [10,11,12,13], i.e., in the humid subtropical (Mediterranean) climate zone characterised by positive yearly air temperatures, high precipitation rates in winter, and dry summers [4].

The presence of *D. reticulatus* ticks in a large area covering various climatic zones suggests their high ecological plasticity, adaptability, and tolerance to temporarily adverse environmental conditions [2]. In periods of negative or extremely high temperature, low relative humidity, or limited access to hosts, ticks enter diapause [14]. In ixodid ticks, diapause can manifest itself in not only inactivity of unfed ticks and metabolism reduced to a minimum, but also a delay in engorgement, a delay in metamorphosis of immature stages and oogenesis in engorged females [15], and even a delay in embryogenesis [16]. This phenomenon is observed in ticks from all climates, in particular those inhabiting the temperate zone [17]. Thanks to the behavioural diapause, ticks can synchronise their questing activity with current conditions and achieve an optimal level of development at a population level [18]. Additionally, with their role as a reservoir and a competent vector, ticks contribute to the maintenance and spread of many pathogens threatening the health and life of animals and humans [2].

Pathogens with the greatest veterinary importance detected in *D. reticulatus* ticks include piroplasmid apicomplexan parasites *Babesia canis*, *B. caballi*, *Theileria equi*, and *Anaplasma marginale* bacteria [2]. *B. canis*, which is the etiological factor of canine babesiosis, should be considered as the most important pathogen transmitted to animals by this tick species. The prevalence of *B. canis* infections in ornate cow ticks collected in different parts of Europe varies significantly. The protozoa has been detected in 0.7% (*n* = 582) of adult *D. reticulatus* ticks in eastern Poland, 1.64% (*n* = 855) in the Netherlands, 3.41% (*n* = 205) in Ukraine, and 14.7% (*n* = 327) in eastern Slovakia [19,20,21]. In turn, *B. caballi* and *T. equi* are the causative agents of equine piroplasmosis, i.e., one of the most commonly diagnosed tick-borne diseases in horses [22]. Bovine anaplasmosis caused by *A. marginale* is a disease of domesticated ruminants worldwide resulting in substantial economic losses [2]. Due to the potential transstadial transmission of *B. canis*, *B. caballi*, and *A. marginale* as well as the transovarial transmission of *B. canis* and *B. caballi*, *D. reticulatus* ticks can serve as a reservoir for these pathogens in the absence of an animal reservoir in the environment [2,23,24,25].

*D. reticulatus* ticks occasionally bite humans [26]. The major viruses transmitted by this species are the tick-borne encephalitis virus (TBEV) [27] causing encephalitis and meningitis in humans, which in extreme cases may lead to paralysis and death, and the phylogenetically closely related Omsk hemorrhagic fever virus (OHFV) [2]. *Rickettsia slovaca* and *R. raoultii* are agents of tick-borne lymphadenopathy (TIBOLA), the first symptom of which is the presence of an eschar on the skin (usually on the head) at the site of tick attachment [28]. The presence of the bacteria *Francisella tularensis* and *Coxiella burnetii* has also been confirmed in *D. reticulatus* ticks, but the role of this species in the transmission of these pathogens in humans is not fully elucidated [2].

Given the widespread distribution of *D. reticulatus* ticks, numerous pathogens transmitted by this species, and the direct negative effects of tick feeding, this species is a real threat to animal and human health. Therefore, it is essential to explore various aspects of tick biology and ecology. This knowledge can help to take measures for minimization of the risk of tick attacks and, consequently, reduce the incidence of tick-borne diseases. To date, research on the seasonal activity of *D. reticulatus* ticks has been conducted in various seasons of the year, including the winter months; however, no studies on the overwintering ability of hungry adult ornate cow ticks in Central Europe, in the natural habitat have been conducted.

The aim of our study was to investigate the ability of adult *D. reticulatus* ticks to overwinter in natural conditions in an endemic area of their occurrence located in the temperate climate zone of eastern Poland. Another objective was to identify factors that affect the survival of ornate cow ticks and analyse the rhythm of their seasonal activity.

## 2. Materials and Methods

Our investigations consisted of the following stages: (i) collection of ticks, (ii) monitoring the survival of *D. reticulatus* adults depending on the prevailing conditions, (iii) determination of the weight of fat bodies in the specimens as one of the indicators of survivability.

### 2.1. Collection of Ticks

Ticks were collected on 5–6 November 2018 in a meadow habitat preferred by this species [2,5]. It represented an unused agricultural land with progressive ecological succession, located in close proximity to buildings within the administrative boundaries of the city of Lublin, eastern Poland (51.2734° N, 22.5345° E). The ticks were collected with the standard flagging method, i.e., sweeping the vegetation with a 1-m^2^ flannel cloth. The specimens were collected simultaneously for 1 h by three people each time. Captured ticks were placed in a plastic container and then transported to the laboratory. Species and their respective development stage and sex were identified using Zeiss STEMI DV4 stereoscopic microscope (Carl Zeiss Light Microscopy, Göttingen, Germany) and the identification guide compiled by Nowak-Chmura [29] were used for identification.

Between the identification of individuals and the next stage of the investigations, the ticks were placed in MEMMERT BE 500 laboratory incubator (Praiston, Leszno, Poland) at a temperature of 10 °C and 75% relative humidity.

### 2.2. Tick Surveillance

The collected hungry *D. reticulatus* adults were assigned into the following groups (i) 240 females and 240 males transferred again to the field for the winter, (ii) 30 females and 30 males kept in laboratory conditions (5 °C, 90% relative humidity), (iii) 30 females and 30 males kept in laboratory conditions (18 °C, 90% relative humidity).

#### 2.2.1. Field Conditions

**Figure 1:**
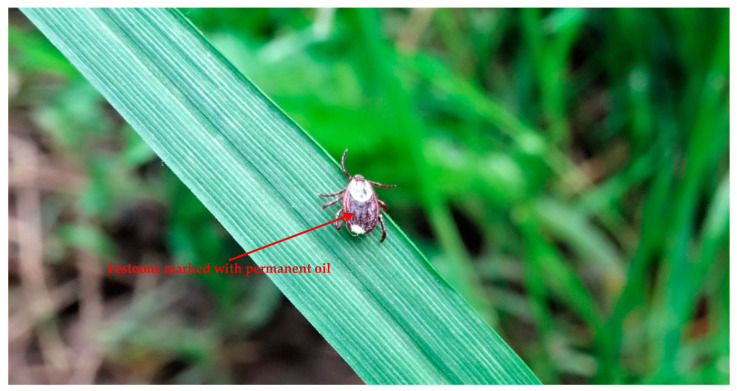
Adult *Dermacentor reticulatus* female in natural condition that previously has been marked with a permanent oil marker (yellow) on the festoons.

Specimens assigned to the first group comprising 240 females and 240 males were marked with a permanent oil marker on the festoons (Figure 1).

**Figure 2:**
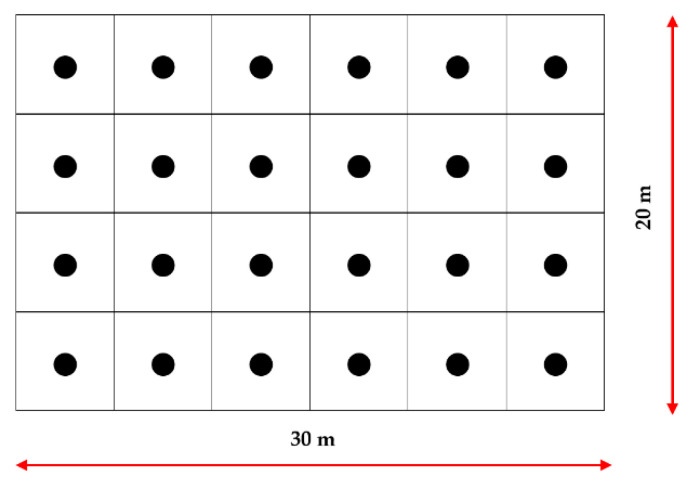
Scheme of the distribution of *Dermacentor reticulatus* ticks in the field, ●—sites of placing the ticks in the experimental plot.

Next, they were transferred to the field on 13.11.2018. At that time, the ambient temperature was 7.0 °C and the relative humidity was 69.1%. In the locality of tick collection (ca. 3-ha fenced area), a 30 × 20 m experimental plot (600 m^2^) was established. The ticks were placed on the plot (on the vegetation, at a height of approx. 10 cm above the ground) at equal spacing (5 m) in groups composed of the same number of specimens (10 females and 10 males in each) (Figure 2). The experimental plot was covered by grassland.

The next stage of the research was conducted after the end of the winter. To determine the largest (real) number of ticks that had survived the winter, the ticks were collected between 25.02 and 4.11.2019 (there was no collection of ticks in summer months, during which ornate cow ticks are inactive in eastern Poland). The flagging method was used and the activity of previously marked specimens was monitored at 7-day intervals. In adverse weather conditions (rainfall, strong wind), ticks were collected at the earliest possible time. During the collection, a white flannel cloth was used to sweep the vegetation. The cloth was inspected after covering a distance of 1 m. Ticks attached to the flag were collected with metal tweezers and placed in a sterile 100-cm^3^ plastic container. At the same time, unmarked individuals showing activity were collected in the experimental plot. Within an hour after the collection, the specimens were transported to the laboratory where captured were stored in a low-temperature ULTF freezer (Arctico, Esbjerg, Denmark) at −80 °C until further determination for the weight of their fat bodies. The species and sex of the unmarked individuals were identified and the specimens were frozen for further use.

Throughout the field study period, the tick collection was accompanied by measurement of current weather conditions, i.e., temperature and relative humidity, using a Data Logger R6030 device (Reed Instruments, Wilmington, NC, USA). Additionally, daily and monthly reports of the Institute of Meteorology and Water Management in Warsaw for Lublin station were used [30].

#### 2.2.2. Laboratory Conditions

On 13.11.2018, another two groups formed from randomly selected *D. reticulatus* ticks, with 30 females and 30 males in each, were transferred into sterile 100-cm^3^ containers. Next, they were placed in MEMMERT BE 500 laboratory incubators (Praiston, Leszno, Poland) with a constant temperature of 5 °C and 18 °C, respectively, 90% relative humidity and 24-h night photoperiod.

In the next stage, the survival of ticks in the laboratory was assessed during 105 observation days. At equal 7-day intervals, the ticks were transferred from the incubators onto a Petri dish for 10 min and then live specimens were checked visually. Dead ticks were placed in special containers and frozen at −80 °C until further analyses. Live specimens were returned to the thermal incubators. This procedure was carried out at room temperature and ca. 75% relative humidity.

### 2.3. Weight of Fat Bodies in Dermacentor reticulatus Ticks

The following groups of 30 females and 30 males were analysed to determine the weight of the fat bodies: (i) randomly selected specimens collected on 5–6 November 2018, (ii) marked ticks that showed activity immediately after overwintering in the field (collected in the period 25 February–18 March 2019), (iii) ticks whose survival was observed in the laboratory conditions, and (iv) marked specimens that showed activity in the last days of field observations in late autumn 2019.

The weight of the fat bodies of the adult *D. reticulatus* ticks was determined with the modified method proposed by Steele and Randolph [31]. The modification consisted of a double-sided puncture of the tick idiosoma with a preparatory needle at the coxa of the fourth pair of legs. This allowed effective penetration of chloroform into the tick’s body and complete dissolution of the fat body. The ticks were weighed using an AS.R2 Plus analytical balance (RADWAG, Radom, Poland) with an accuracy of 0.01 mg.

### 2.4. Statistical Analysis

The statistical tests were chosen based on the characteristics of the distribution of the analysed variables with the use of the Lilliefors test.

To determine the significance of differences between the seasonal activity of ticks that overwintered in the field (marked ticks) and the seasonal activity of the local *D. reticulatus* population (dependent samples), the two-tailed *t* test was used (normal distribution). The effect of weather conditions (temperature and relative air humidity) prevailing at the time of collection on tick activity was checked using rho-Spearman correlations.

The survival rates of the *D. reticulatus* ticks kept in the laboratory were compared with a test assessing the significance of the difference between two structure indicators. The differences in the weight of the fat bodies between the examined groups: ticks kept at 5 °C and 18 °C, ticks kept in laboratory conditions and overwintering (active in spring 2019), ticks kept in laboratory conditions and overwintering (active in late autumn 2019), ticks collected in late autumn 2018 and ticks overwintered collected in early spring 2019, overwintered ticks collected in the field in early spring 2019, and overwintered ticks collected in late autumn 2019 were analysed using the ANOVA Kruskal-Wallis test with Bonferroni-Dunn correction (adjusted for ties).

The value of *p* < 0.05 was considered statistically significant. Statistical calculations were performed using the STATISTICA 10 PL statistical package (StatSoft, TIBCO Software Inc, Palo Alto, CA, USA).

## 3. Results

### 3.1. Overwintering of Dermacentor reticulatus Ticks in Natural Conditions

Among the 480 (240 females and 240 males) adult *D. reticulatus* ticks, 163 females (67.9%) and 144 males (60.0%) survived the winter and showed activity on the consecutive measurement days (excluding specimens that either found a host or did not survive the winter) (Table 1). Various numbers of active ticks were noted between February and November, excluding the summer months. The observed rhythm of seasonal activity of ticks that had survived the winter did not differ significantly (*t* = −0.6889, *p* = 0.4996) from the general activity of the *D. reticulatus* population co-occurring in the examined locality. In both cases, a higher number of ticks were active in spring than in autumn. Females dominated the active specimens (Table 1).

During the *D. reticulatus* overwintering period in the natural conditions, January was the coldest month with an average air temperature of −3.4 °C. Negative mean minimum temperature values were also recorded in November, December, and February. In December and January, the mean maximum temperature did not exceed 5 °C. The highest precipitation rates were recorded in the winter months, whereas the summer was characterised by the lowest rainfall. The persistence of the snow cover varied from December to April (Table 2).

During the field study, *D. reticulatus* ticks were collected at a temperature range of 7.0–22.9 °C and 61–86.7% relative humidity (Table 1). A statistically non-significant negative correlation was found between the relative air humidity and the number of active ticks that survived the winter (r_s_ = −0.4222, *p* = 0.8637). There was also a statistically significant negative correlation between the temperature and tick activity (r_s_ = −0.4951, *p* = 0.0311).

### 3.2. Survivability of Dermacentor reticulatus Ticks in Laboratory Conditions

During the 105 observation days, the survival rate of *D. reticulatus* adults ranged from 73% to 83%. The greatest number of ticks survived at 18 °C. In these conditions, 100% females and 97% males survived during the first 56 days of observation, and their survival remained at this level until day 70. Higher mortality was observed in the group of females kept at 5 °C (Table 3). There was no statistically significant difference in the survival rates between the *D. reticulatus* groups kept at 5 °C and 18 °C (depending on the day of observation, the probability level ranged from *p* = 1.00000 to *p* = 0.1068).

### 3.3. Weight of Fat Bodies in Dermacentor reticulatus Ticks

The average weight of the fat body in *D. reticulatus* ticks collected in autumn 2018 was 0.28 ± 0.13 mg (females) and 0.29 ± 0.21 mg (males). In ticks that were active immediately after overwintering, these values were 0.30 ± 0.15 mg (females) and 0.27 ± 0.15 mg (males). In comparison with these specimens, ticks that had survived the winter and exhibited questing activity until late autumn 2019 had lower average weight of fat bodies (females 0.19 ± 0.08 mg; males 0.23 ± 0.11 mg) (Table 4, Supplement S1).

There was no significant difference in the weight of the fat bodies between *D. reticulatus* ticks kept at 5 °C and 18 °C (H = 0.0831, *p* = 0.7730). There was no statistically siginificant difference in the fat body weight between ticks kept in laboratory conditions and specimens overwintering in the field (active in spring 2019) (H = 0.5576, *p* = 0.4551). Similarly, no such relationship was found between ticks collected in late autumn 2018 exhibiting spring activity after overwintering (H = 0.005, *p* = 0.9410).

A statistically significant difference in the weight of the fat bodies was found between marked ticks that had overwintered in the field (collected in early spring 2019) and marked ticks active in late autumn 2019 (H = 5.3245, *p* = 0.0210).

## 4. Discussion

The *D. reticulatus* survivability depends mainly on the thermal conditions, relative humidity, and availability of potential hosts [2]. The dynamically changing weather conditions associated with the progressing global warming may have a significant impact on the number, occurrence range, and dynamics of the seasonal rhythms of ticks [32], e.g., ornate cow ticks, and may indirectly contribute to an increase in the incidence of some tick-borne diseases in humans [33].

In the area examined in the present study, the average air temperature has changed from 16.7 °C to 18.0 °C in summer and from −2.5 °C to −0.5 °C in winter over the last 30 years [34,35]. As shown by our results, adult *D. reticulatus* with survival rates of 67.9% of females and 60.0% of males (Table 1), successfully survive winter with a majority of their population. The value of the survival indicator should be expected to grow in the case of a further increase in average temperatures. In the more severe climate of the European part of Russia, *D. reticulatus* ticks can survive for 2.5 years [36] or even up to 4 years, as reported by Olsuf’ev [37]. Engorged larvae, nymphs, and females of the ornate cow tick are also able to survive the period of frosty winter in natural conditions [38]. The latter are then able to lay eggs in spring, from which larvae hatch [39]. A consequence of the increasingly warmer seasons of the year may be the predominance of the *D. reticulatus* species, which in contrast to other ixodid ticks widespread in the temperate climate zone, e.g., *Ixodes ricinus*, is active in a wider temperature range and at lower relative air humidity [40]. It has been confirmed in laboratory and field studies that *D. reticulatus* adults can survive 150 days at −10 °C [41] and 100 days of flooding [42].

In the temperate climate zone, *D. reticulatus* adult generally exhibit year-round activity with periods of fluctuating intensity [2]. In different localities, the highest ornate cow tick activity is noted in spring (e.g., Russia, northern Poland) [36,43] or in autumn (e.g., eastern Poland) [44,45] and the same population observed over a long period may exhibit alternating activity peaks [5]. In Poland, *D. reticulatus* ticks were collected from vegetation in winter periods characterised by a rise in air temperature up to 5–10 °C [46,47]; obviously, the number of active specimens at that time was significantly lower than in spring or autumn [48]. In the present study, temperatures over 5 °C (facilitating questing activity) in the winter months were recorded on 9 days in November and 15 days in February (Table 2). The results of our study showed that the survival rate of the adult ornate cow ticks in laboratory conditions (5 °C and 18 °C; 90% RH) during 105 days of observation did not differ significantly and reached 80% of males and 73% of females (Table 3). Furthermore, the analysed groups did not differ significantly in their fat body weight. In contrast, there was a significant difference in this parameter between the laboratory specimens and the group of overwintering ticks showing host-questing activity in the following season (late autumn) (Table 4, Supplement S1). This demonstrates that, in laboratory conditions (mimicking natural conditions in which ticks display host-seeking activity) in the absence of external stimuli, e.g., the smell and mobility of animals, changing weather conditions, or sunlight, *D. reticulatus* ticks enter diapause, as at negative temperatures [14].

Fat bodies, which serve as an energy reserve, allow physiological processes, locomotion, and questing activity. For instance, *I. ricinus* nymphs utilise 0.00018 mg of fat per day [31]. The fat body determines the lifespan in ticks [49], as it supports long survival in nature, even up to 4 years without access to hosts, as in the case of *D. reticulatus* [37]. Based on the classification of the physiological age of ticks proposed by Razumov [49], which takes into consideration the weight and distribution of fat bodies in the population, the analysed *D. reticulatus* population can be classified as “mature”. This is confirmed by our previous investigations of the host-questing activity of ticks conducted in the same area (Plot C) [50]. The analysed population of ornate cow ticks has displayed repeated activity. This was associated with the specific location of the examined urban site and limited access to medium-sized and large animals. Therefore, it can be assumed that the survival rate estimated at 69.7% in females and 60.0% in males in the present study is burdened with a slight error caused by the migration of specimens that found a host during the study period. Noteworthy is the greater survival of females despite the equal numbers of both sexes used in the experiment. This is probably related to their higher survivability [51].

## 5. Conclusions

The present study shows that, on the population scale, adult *Dermacentor reticulatus* ticks are able to survive winter in temperate climate conditions at a level ensuring a subsequent increase in the population size.

Fat bodies are energy reserves used by ornate cow ticks for host-questing activity. The absence of a statistically significant difference between the fat body weights in the ticks that were active before the winter and the wintering ticks indicates that in the case of adverse weather conditions (frost, snow), adult *D. reticulatus* ticks enter diapause. In laboratory conditions (mimicking natural conditions in which ticks display host-seeking activity) in the absence of external stimuli, e.g., the smell and mobility of animals, or sunlight, *D. reticulatus* ticks enter diapause, as at negative temperatures, thus maximally limiting energy consumption. This allows them to survive in the environment for a long time waiting for a potential host.

The high overwintering rate may be one of the main causes of the *D. reticulatus* expansion to new areas and the increase in the number of this species observed in recent years. Given their high epidemiological importance, the research into the biology and ecology of ornate cow ticks should be continued.

## Figures and Tables

**Table 1:** Seasonal activity of *Dermacentor reticulatus* individuals assessed after overwintering and a local population of this species co-occurring in the examined site.

Season of the Year	Date	Weather Conditions at the Time of Collection		Number of Active Specimens (Co-Occurring)		Number of Active Specimens (after Overwintering)	
		T [⁰C]	H [%]	F	M	F (*n* = 240)	M (*n* = 240)
**Spring 2019**	25.02	08.9	77.0	8	6	12	14
	11.03	07.0	81.0	11	9	9	10
	18.03	13.3	70.5	9	10	22	16
	25.03	13.5	67.1	13	10	18	19
	01.04	20.1	65.0	7	9	14	17
	08.04	16.0	80.0	8	6	10	8
	15.04	22.5	61.1	12	11	5	9
	23.04	17.0	61.0	9	6	10	5
	30.04	15.1	79.0	9	7	11	8
	08.05	22.5	73.0	4	2	3	1
	15.05	22.9	70.1	5	0	2	2
**Autumn 2019**	02.09	20.0	72.9	6	3	2	0
	16.09	20.5	74.4	11	5	1	5
	23.09	18.0	79.8	9	8	10	8
	30.09	16.1	80.1	11	5	17	12
	07.10	20.3	80.2	11	3	8	2
	14.10	17.3	68.9	8	3	5	7
	25.10	18.0	62.0	13	5	3	1
	04.11	11.0	86.7	6	1	1	0
**Total**				170	109	163	144

T—temperature, H—relative air humidity, F—females, M—males, *n*—number of ticks used in the experiment.

**Table 2:** Weather conditions recorded by Lublin station during the study on the survivability of *Dermacentor reticulatus* ticks; data from the Institute of Meteorology and Water Management [30].

Month	Weather Parameters						
	T_AV_ [°C]	T_MAX_ [°C]	T_MIN_ [°C]	H [%]	PP [mm]	SN [days]	T_MAX_ < 5 °C [days]
11.2018 *	0.3	3.0	−2.2	88.4	6.6	0	9
12.2018	0.1	2.1	−2.2	91.3	65.5	14	0
01.2019	−3.4	−1.1	−6.3	87.6	47.5	26	0
02.2019	2.0	5.7	−1.1	80.3	18.0	5	15
03.2019	4.9	9.8	0.8	71.6	23.0	5	30
04.2019	9.3	14.8	4.0	58.3	31.0	1	30
0.52019	13.5	18.0	12.3	77.0	26.0	0	31
06.2019	21.3	27.3	15.0	68.3	29.0	0	30
07.2019	19.4	24.5	12.0	64.4	31.0	0	31
08.2019	19.7	25.9	13.8	68.2	28.0	0	31
09.2019	14.1	19.6	9.1	75.7	46.5	0	30
10.2019	10.5	16.2	6.2	81.3	35.0	0	31
11.2019 *	7.6	12.2	2.7	81.2	7.3	0	2

*—the analysis only included days when the ticks stayed in the field, T_AV_—average temperature, T_MAX_—average maximum temperature, T_MIN_—average minimum temperature, H—average relative humidity, PP—total rainfall and/or snowmelt, SN—snow indicator (in the monthly average, total days with snow).

**Table 3:** Survival rates in adult *Dermacentor reticulatus* ticks in laboratory conditions in the period of 13 November 2018–25 February 2019; the observations were carried out at equal 7-day intervals.

Day	Number of Live Specimens (*n* = 120)			
	5 °C; 90% RH		18 °C; 90% RH	
	F (%)	M (%)	F (%)	M (%)
7	30 (100)	30 (100)	30 (100)	30 (100)
14	30 (100)	30 (100)	30 (100)	30 (100)
21	30 (100)	30 (100)	30 (100)	30 (100)
28	30 (100)	30 (100)	30 (100)	30 (100)
35	30 (100)	29 (97)	30 (100)	30 (100)
42	30 (100)	28 (93)	30 (100)	29 (97)
49	28 (93)	28 (93)	30 (100)	29 (97)
56	28 (93)	28 (93)	30 (100)	29 (97)
63	25 (83)	28 (93)	28 (93)	29 (97)
70	25 (83)	24 (80)	26 (87)	29 (97)
77	25 (83)	24 (80)	25 (83)	27 (90)
84	25 (83)	24 (80)	25 (83)	27 (90)
91	24 (80)	24 (80)	25 (83)	25 (83)
98	22 (73)	24 (80)	25 (83)	24 (80)
105	22 (73)	24 (80)	25 (83)	24 (80)

F—females, M—males, n—number of observed specimens, RH—relative air humidity.

**Table 4:** Mean weight of fat bodies of adult *Dermacentor reticulatus* ticks in each group of females and males.

Parameter	Mean Weight of Fat Bodies of Analysed Specimens [mg]									
	Laboratory Conditions *(n = 30)*				Field Conditions (*n* = 30)					
	5 °C		18 °C		Autumn 2018		Spring 2019		Autumn 2019	
	F	M	F	M	F	M	F	M	F	M
M	0.25	0.28	0.26	0.27	0.28	0.29	0.30	0.27	0.19	0.23
SD	0.11	0.12	0.12	0.16	0.13	0.21	0.15	0.15	0.08	0.11
Med	0.26	0.27	0.25	0.24	0.27	0.26	0.26	0.27	0.18	0.21
Min	0.07	0.09	0.06	0.07	0.08	0.04	0.05	0.07	0.05	0.05
Max	0.52	0.55	0.53	0.65	0.67	0.80	0.66	0.70	0.37	0.45

M—Mean, SD—Standard deviation, Med—median, Min—minimum value, Max—maximum value, F—Females, M—Males, *n*—number of females and males in each group.

## Supplementary Materials

The following are available online at https://www.mdpi.com/2079-7737/9/7/145/s1, Supplement S1: Weight of fat bodies in *Dermacentor reticulatus* ticks.
